# Ginsenoside Re Improves Isoproterenol-Induced Myocardial Fibrosis and Heart Failure in Rats

**DOI:** 10.1155/2019/3714508

**Published:** 2019-01-01

**Authors:** Quan-wei Wang, Xiao-feng Yu, Hua-li Xu, Xue-zhong Zhao, Da-yuan Sui

**Affiliations:** ^1^Departments of Cardiovascular Medicine, First Hospital, Jilin University, Changchun 130021, China; ^2^Departments of Pharmacology, College of Pharmacy, Jilin University, Changchun 130021, China

## Abstract

*Objective. Panax ginseng* is used widely for treatment of cardiovascular disorders in China. Ginsenoside Re is the main chemical component of* P. ginseng*. We aimed to investigate the protective effect of ginsenoside Re on isoproterenol-induced myocardial fibrosis and heart failure in rats.* Methods.* A model of myocardial fibrosis and heart failure was established by once-daily subcutaneous injection of isoproterenol (5 mg/kg/day) to rats for 7 days. Simultaneously, rats were orally administrated ginsenoside Re (5 or 20 mg/kg) or vehicle daily for 4 weeks.* Results*. Isoproterenol enhanced the heart weight, myocardial fibrosis, and hydroxyproline content in rat hearts. Ginsenoside Re inhibited (at least in part) the isoproterenol-induced increase in heart weight, myocardial fibrosis, and hydroxyproline content. Compared with the isoproterenol group, treatment with ginsenoside Re ameliorated changes in left ventricular systolic pressure, left ventricular end diastolic pressure, and the positive and negative maximal values of the first derivative of left ventricular pressure. Ginsenoside Re administration also resulted in decreased expression of transforming growth factor (TGF)-*β*1 in serum and decreased expression of Smad3 and collagen I in heart tissue.* Conclusion.* Ginsenoside Re can improve isoproterenol-induced myocardial fibrosis and heart failure by regulation of the TGF-*β*1/Smad3 pathway.

## 1. Introduction

Myocardial ischemia is a cardinal cardiovascular disease and a major cause of morbidity and mortality worldwide [1]. Ischemic injury leads to the development of myocardial fibrosis due to the negligible regenerative capacity of the heart. Myocardial fibrosis is associated with increased deposition of matrix proteins in the myocardium.

Myocardial fibrosis can be divided into “reactive interstitial” and “reparative” subtypes [2]. Reactive interstitial fibrosis is characterized by expansion of the myocardial interstitial space without significant loss of cardiomyocytes. Reparative fibrosis involves collagen deposition in response to myocardial ischemia. The adaptive responses to preserve cardiac output are reactive interstitial fibrosis and cardiomyocyte hypertrophy. Eventually, cardiomyocytes undergoing necrosis and apoptosis cause reparative fibrosis [3]. Myocardial fibrosis plays an important part in the pathogenesis of ischemic cardiomyopathy, which contributes to systolic and diastolic dysfunction. Ischemic injury to the heart leads to myocardial fibrosis, and the end result is heart failure.

Traditional Chinese medicine (TCM) has been used widely in China, Korea, Japan, and other Asian countries for the treatment of cardiovascular diseases. Furthermore, an increasing number of studies have shown that TCM can improve the outcome in the treatment of ischemic heart disease [4].

The TCM* Panax ginseng* belongs to the Araliaceae family and is distributed in 35 countries, particularly in China and South Korea.* P. ginseng* has been used widely in China for thousands of years against various diseases. There is increasing evidence of the beneficial effect of* P. ginseng* on cardiac disease [5]. Tsai and colleagues showed that, after* P. ginseng* treatment in rats suffering from diabetes mellitus (DM) induced by streptozotocin, cardiac output was found to be enhanced markedly.* P. ginseng* also increased expression of peroxisome proliferator-activated receptor (PPAR)*δ* in the hearts of DM rats. Therefore, Tsai and colleagues suggested that* P. ginseng* could improve heart failure by increasing PPAR*δ* activity in DM rats [6]. In a rat model with ligation of coronary arteries,* P. ginseng* reversed cardiac hypertrophy, myocardial remodeling, and heart failure by inhibition of calcineurin activation [7].

Ginsenosides are the major pharmacologic ingredients of* P. ginseng*, and several have been isolated and identified. Depending on their structures, ginsenosides can be divided into three groups: “panaxatriol” (Re, Rf, Rg1, Rg2, and Rh1), “panaxadiol” (Rb1, Rb2, Rb3, Rc, Rd, Rg3, and Rh2) and oleanolic acid [8]. Sui and coworkers showed that ginsenoside Rg3 improves cardiac function in rats undergoing myocardial ischemia–reperfusion [9] and that ginsenoside Rh1 attenuates myocardial injury and enhances heart function in rats given isoproterenol [10]. Re is the major ginsenoside in* P. ginseng* extract. The pharmacokinetics of ginsenoside Re have revealed that the time needed to reach the peak plasma concentration after oral administration is 0.4 ± 0.2 h and that its oral bioavailability is 0.19%–0.28% [11].

In the present study, we wished to investigate the effect of ginsenoside Re on isoproterenol-induced myocardial fibrosis and heart failure in rats.

## 2. Methods

### 2.1. Ethical Approval of the Study Protocol

The study protocol was approved by the Ethics Committee of Jilin University (Changchun, China). Experiments were carried out according to the* Guidelines for the Care and Use of Laboratory Animals* (publication 86-23, revised in 1986; National Institutes of Health (NIH), Bethesda, MD, USA).

### 2.2. Animals

Male Wistar rats (8–10 weeks; 260–280 g) were provided by the Experimental Animal Center of Norman Bethune University of Medical Science (Changchun, China). Animals were housed in diurnal lighting conditions (12 h/12 h) and allowed free access to food and water for 7 days before experimentation.

### 2.3. Chemical Reagents

Ginsenoside Re (purity = 98.6%) was from Professor Yanping Chen in Jilin University. A Sirius Red staining kit was purchased from Solarbio Life Sciences (Beijing, China). A hydroxyproline kit was obtained from Nanjing Jiancheng Bioengineering Institute (lot number 20160109; Nanjing, China). ELISA kits for transforming growth factor (TGF)-*β*1 (product code ab119558), rabbit anti-collagen I, rabbit anti-Smad3, and rabbit antiphosphorylated (p)-Smad3 were obtained from Abcam (Cambridge, UK). Isoproterenol was purchased from Sigma–Aldrich (Saint Louis, MO, USA). Carboxymethyl cellulose sodium salt (CMC-Na) was from Shanghai Jinshan Chemicals (Shanghai, China). Protein-extraction kits, rabbit anti-*β*-actin antibody, and an electrochemiluminescence (ECL) kit were from Beyotime Institute of Biotechnology (Jiangsu, China).

### 2.4. Experimental Protocol

**Figure 1:**
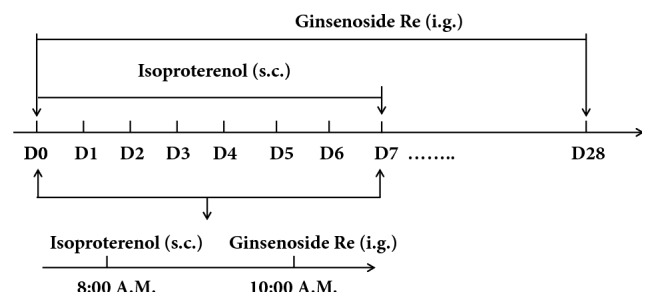
Experimental design.

According to previous study [12], the doses of ginsenoside at 5, 10, and 20 mg/kg are good in rat model. Therefore, we chose the dose of ginsenoside Re at 5 and 20 mg/kg. Sixty rats were divided randomly into four groups of 15: control, isoproterenol, and ginsenoside Re (5 and 20 mg/kg) groups. The mortality of animals in control, isoproterenol, and ginsenoside Re (5 and 20 mg/kg) groups was 1, 3, 3, and 2, respectively. Isoproterenol was dissolved in physiologic saline (0.9%) and injected (5 mg/kg body weight, s.c.) once-daily for 7 consecutive days to induce myocardial fibrosis and heart failure [13]. Rats in control and isoproterenol groups were administered 0.5% CMC-Na once-daily for 4 weeks. Rats in ginsenoside-Re groups were treated with ginsenoside Re (5 or 20 mg/kg) once-daily for 4 weeks (Figure 1).

### 2.5. Measurement of Cardiac Function

Twenty-four hours after the final administration of ginsenoside Re or CMC-Na, rats were anesthetized with urethane (1 g/kg, i.p.). The right common carotid artery was cannulated with a 2-F polyethylene catheter into the left ventricle. Then, left ventricular end diastolic pressure (LVEDP), left ventricular systolic pressure (LVSP), and positive (+dp/dt) and negative (−dp/dt) maximal values of the first derivative of left ventricular pressure were measured using a hemodynamic analyzing system (RM-6000; Nihon Kohden, Japan).

### 2.6. Assessment of Indices of Heart Weight

After measurement of cardiac function, rats were sacrificed with carbon dioxide. The thoracic cavity was opened to expose the heart. The heart was removed rapidly and heart tissues washed with cold phosphate-buffered saline. The heart was weighed after separating the atria, aorta, and adipose tissue. The body weight of the rat was also recorded. The heart weight index (HWI, in mg/g) was calculated by dividing the heart weight by body weight.

### 2.7. Histopathology

Heart tissues (3 rats in each group) were fixed in 10% formalin solution. Specimens were embedded in paraffin and then cut into 5 *μ*m thick sections. After staining with Sirius Red according to manufacturer instructions, sections were examined by an experienced observer blinded to the treatment protocol using a microscope (IX-70; Olympus, Tokyo, Japan). Ten fields were selected randomly from five sections in each heart and the images of myocardial sections stored. To calculate the ratio of the area of interstitial fibrosis in the heart, images were imported into NIH-Image software, which was used for quantitative analyses. The percentage of fibrotic tissue in the heart was calculated using the following formula: Fibrotic tissue in the heart (%) = (fibrotic tissue area)/(fibrotic tissue area + cardiomyocyte area) × 100%

### 2.8. Determination of Hydroxyproline

Hydroxyproline content in hearts was determined using a commercial kit in accordance with manufacturer instructions. Hydroxyproline content was expressed in milligrams per total weight of heart tissue.

### 2.9. TGF-*β*1 Assay

Blood samples were collected after measurement of cardiac function. Samples were centrifuged (1500 × *g*) for 15 min at 4°C. Serum was harvested and the TGF-*β*1 level in serum assayed according to manufacturer instructions.

### 2.10. Western Blotting

Proteins from hearts were loaded (50 *µ*g) and subjected to sodium dodecyl sulfate-polyacrylamide gel (10%) electrophoresis. Proteins were transferred to polyvinylidene fluoride (PVDF) membranes (Millipore, Bedford, MA, USA) for 2 h at 110 V. After blockade with 5% nonfat milk for 2 h, PVDF membranes were incubated with primary antibodies overnight at 4°C: rabbit anti-collagen I (1:1000 dilution), rabbit anti-Smad3 (1:2000), rabbit anti-p-Smad3 (1:1500), or rabbit anti-*β*-Actin (1:1500). PVDF membranes were processed with horseradish peroxidase-labeled secondary antibody (1:3000 dilution). Bands were visualized using ECL detection reagents (Beyotime Institute of Biotechnology). For quantitative analyses of band density, densitometry was done with Gel-Pro® Analyzer v3.0. The ratio of the density of bands of the detected protein to that of *β*-actin was used for statistical analyses.

### 2.11. Statistical Analyses

Data are the mean ± SD. Analyses were done using SPSS v22.0 (IBM, Armonk, NY, USA). Data were analyzed with one-way analysis of variance followed by Tukey's* post hoc* test.* P* < 0.05 was considered significant.

## 3. Results

### 3.1. Effect of Ginsenoside Re on Myocardial Hypertrophy and the HWI

**Figure 2:**
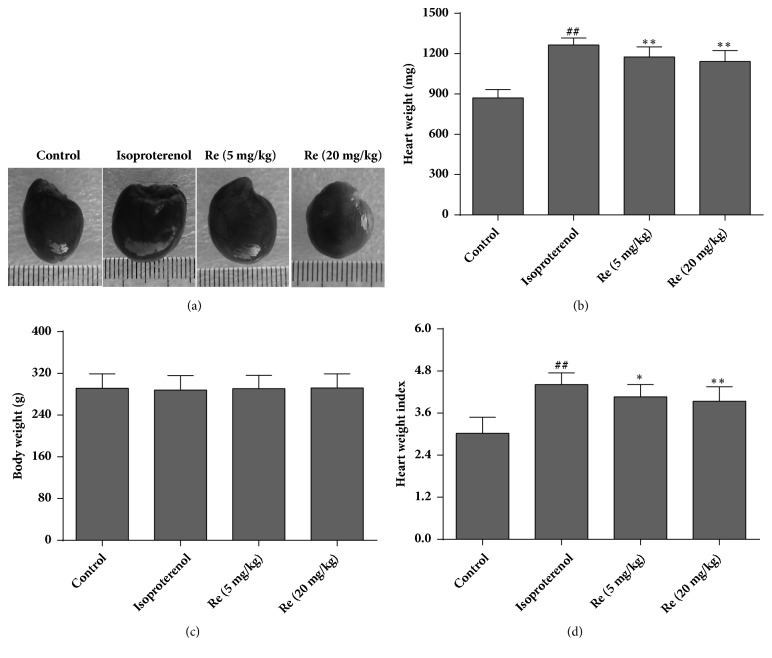
Effect of ginsenoside Re on myocardial hypertrophy and the heart weight index in isoproterenol-induced myocardial fibrosis and heart failure in rats. (a) Representative hearts of rats in each of the four groups, (b) heart weight, (c) body weight, and (d) heart weight index. Data are the mean ± SD. Control group: n = 14; isoproterenol group: n = 12; ginsenoside Re (5 mg/kg) group: n = 12; ginsenoside Re (20 mg/kg) group: n = 12. ^##^*P *< 0.01 compared with the control group. ^*∗*^*P *< 0.05 or ^*∗∗*^*P *< 0.01 compared with the isoproterenol group.

Rat hearts showed marker hypertrophy in the isoproterenol group (Figure 2(a)). However, treatment with ginsenoside Re attenuated the myocardial hypertrophy induced by isoproterenol. Significant differences in body weight were not observed among the four groups (*P* > 0.05) (Figure 2(c)). Heart weight (mg) and the HWI (mg/g) increased in the isoproterenol group (1264.1±53.1 and 4.41±0.33) as compared with the control group (870.0±63.5 and 3.02±0.46), respectively. Compared with the isoproterenol group, the heart weight and HWI in the ginsenoside Re (5 mg/kg) group (1175.8±75.0 and 4.06±0.35) and ginsenoside Re (20 mg/kg) group (1142.1±80.6 and 3.94±0.41), respectively, were reduced significantly (*P *< 0.05 or* P* < 0.01) (Figures 2(b) and 2(d)).

### 3.2. Effect of Ginsenoside Re on Cardiac Function

**Figure 3:**
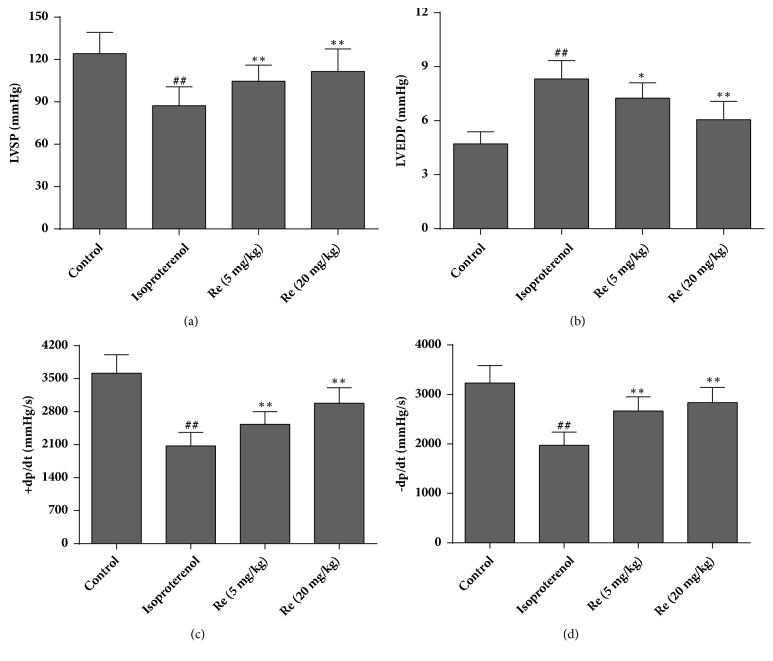
Effect of ginsenoside Re on cardiac function in isoproterenol-induced myocardial fibrosis and heart failure in rats. (a) Left ventricular systolic pressure (LVSP), (b) left ventricular end diastolic pressure (LVEDP), (c) +dp/dt, and (d) −dp/dt. Data are the mean ± SD. Control group: n = 14; isoproterenol group: n = 12; ginsenoside Re (5 mg/kg) group: n = 12; ginsenoside Re (20 mg/kg) group: n = 12. ^##^*P* < 0.01 compared with the control group. ^*∗*^*P* < 0.05 or ^*∗∗*^*P* < 0.01 compared with the isoproterenol group.

Isoproterenol caused reductions in ±dp/dt and LVSP and a significant increase in LVEDP, as compared with rats in the control group (*P* < 0.01). Changes in these parameters suggested that isoproterenol induced impairment in cardiac function in rats. Administration of ginsenoside Re (5 or 20 mg/kg) for 4 weeks to isoproterenol-challenged rats alleviated the reductions in ±dp/dt and LVSP and increase in LVEDP markedly (*P* < 0.05 or* P* < 0.01, Figure 3). These findings suggested that ginsenoside Re could improve systolic and diastolic function after myocardial ischemia.

### 3.3. Effect of Ginsenoside Re on Myocardial Fibrosis

**Figure 4:**
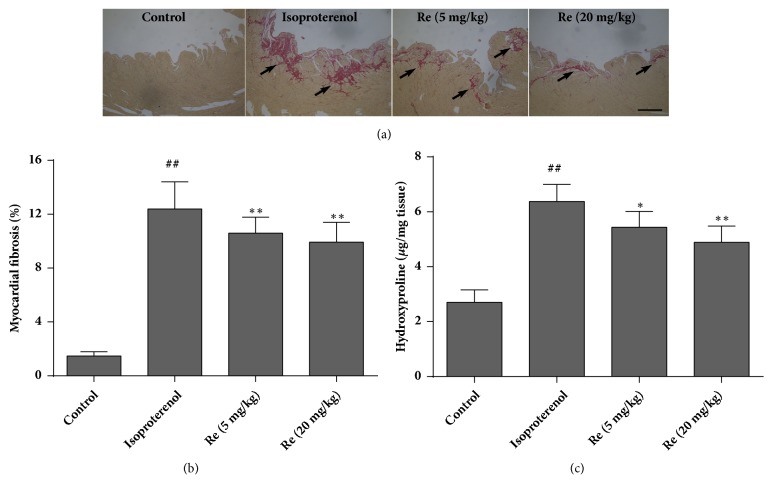
Effect of ginsenoside Re on myocardial fibrosis and hydroxyproline content in isoproterenol-induced myocardial fibrosis and heart failure in rats. (a) Representative photomicrographs of Sirius Red staining of rat hearts. Collagen fibers were stained red (black arrows) and myocardial tissues were stained yellow (n = 3), (b) bar graph of myocardial fibrosis (percentage of fibrotic tissue in hearts), and (c) bar graph of hydroxyproline content. Control group: n = 8; isoproterenol group: n = 6; ginsenoside Re (5 mg/kg) group: n = 6; ginsenoside Re (20 mg/kg) group: n = 7. Data are the mean ± SD. ^##^*P* < 0.01 compared with the control group. ^*∗*^*P* < 0.05 or ^*∗∗*^*P* < 0.01 compared with the isoproterenol group. Bar=100 *μ*m.

Sirius Red staining is a well-established method to assess myocardial fibrosis. Collagen fibers are stained red, whereas myocardial tissues are stained yellow. Rat hearts in the control group were stained with a few red-stained tissues. However, rats' hearts of the isoproterenol group showed a marked increase in the number of collagen fibers as compared with rat hearts in the control group. Treatment with ginsenoside Re reduced the augmentation of collagen fibers induced by isoproterenol (Figure 4(a)). The red-stained area was also calculated as a percentage of the total area using Image-Pro Plus software (Media Cybernetics, Silver Spring, MD, USA). The red-appearing collagen areas in rat hearts were significantly higher in isoproterenol-challenged rats (*P* < 0.01). The number of collagen fibers in the hearts of rats from the ginsenoside-Re groups was reduced (*P* < 0.01) (Figure 4(b)). Hydroxyproline is also a marker of fibrosis because of its restricted and unique distribution in collagen. In accordance with the results of Sirius Red staining, isoproterenol resulted in a significant increase in hydroxyproline content in hearts as compared with that of rats in the control group. Compared with the isoproterenol group, administration of ginsenoside Re (5 or 20 mg/kg) reduced hydroxyproline content in rat hearts by 14.7% or 23.3%, respectively (*P* < 0.05 or* P* < 0.01) (Figure 4(c)).

### 3.4. Effect of Ginsenoside Re on the TGF-*β*1 Level

**Figure 5:**
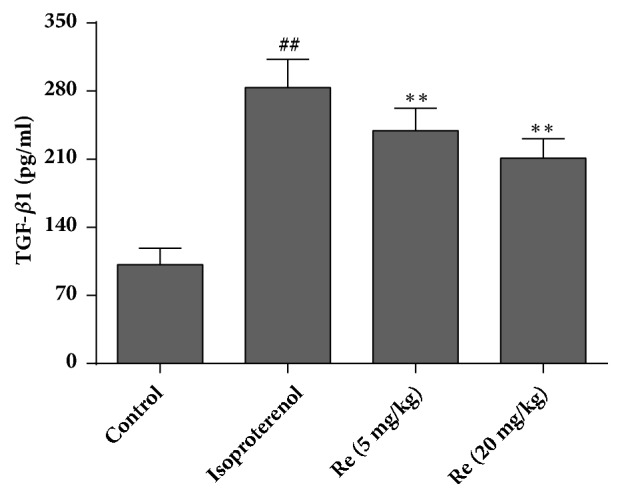
Effect of ginsenoside Re on TGF-*β*1 expression in isoproterenol-induced myocardial fibrosis and heart failure in rats. Data are the mean ± SD. Control group: n = 14; isoproterenol group: n = 12; ginsenoside Re (5 mg/kg) group: n = 12; ginsenoside Re (20 mg/kg) group: n = 12. ^##^*P* < 0.01 compared with the control group. ^*∗∗*^*P* < 0.01 compared with the isoproterenol group.

The TGF-*β*1 level in rat serum was measured. Compared with the control group (101.7±16.9 pg/mL), the TGF-*β*1 level was augmented significantly in the isoproterenol group (283.6±29.2 pg/mL) (*P* < 0.01). Treatment with ginsenoside Re (5 or 20 mg/kg) attenuated the increase in the TGF-*β*1 level (239.2±23.1 and 210.9±20.2 pg/mL, respectively) (*P* < 0.01) (Figure 5).

### 3.5. Effect of Ginsenoside Re on Expression of p-Smad3 and Collagen I

**Figure 6:**
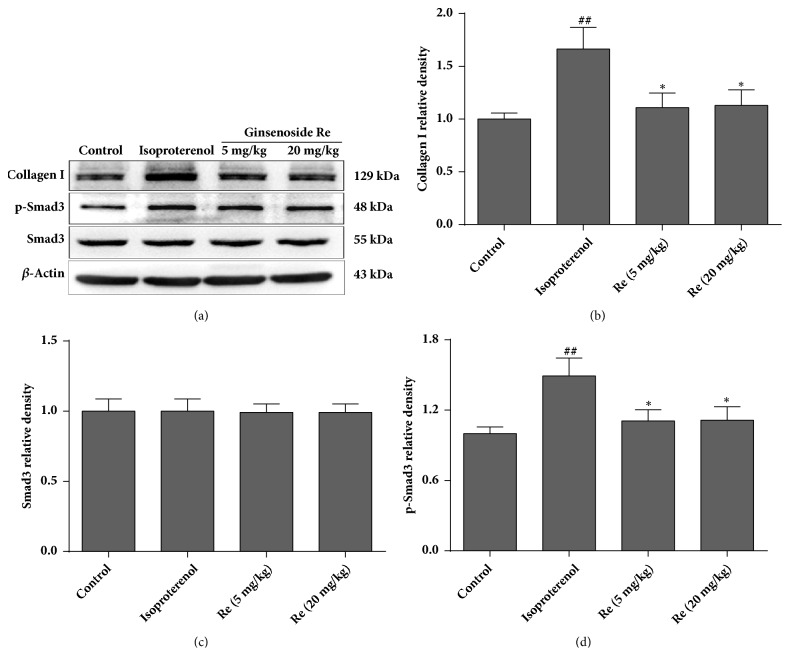
Effect of ginsenoside Re on expression of p-Smad3 and collagen I in isoproterenol-induced myocardial fibrosis and heart failure in rats. (a) Representative western blots of collagen I, p-Smad3 and Smad3, (b) bar graphs of collagen-I expression, (c) bar graphs of Smad3 expression, and (d) bar graphs of p-Smad3 expression. Data are the mean ± SD (n = 3). ^##^*P* < 0.01 compared with the control group. ^*∗*^*P* < 0.05 compared with the isoproterenol group.

Figure 6 shows the effect of ginsenoside Re on expression of p-Smad3 and collagen I in rat hearts. No differences were observed in Smad3 expression among groups. Expression of p-Smad3 and collagen I in the isoproterenol group increased markedly as compared with that of the control group (*P* < 0.01). However, after treatment with ginsenoside Re (5 or 20 mg/kg), expression of p-Smad3 and collagen I decreased significantly (*P* < 0.05).

## 4. Discussion

The present study suggested that treatment with ginsenoside Re suppressed isoproterenol-induced myocardial fibrosis and heart failure markedly in rats. The pharmacologic actions of ginsenoside Re could be attributed to regulation of the TGF-*β*1/Smad3 pathway.

Isoproterenol is a *β*-adrenergic agonist. The model of heart failure induced by isoproterenol in animals has been used widely to evaluate the protective effects of several agents on heart dysfunction caused by myocardial ischemia [14, 15]. The pathophysiologic and morphologic alterations of this noninvasive model are comparable with those of heart failure in humans [16].

Several scholars have reported that isoproterenol-induced impairment of heart function is characterized by increased LVEDP and decreased LVSP as well as ±dp/dt [17]. To evaluate the effect of ginsenoside Re on heart failure following myocardial ischemia, hemodynamic parameters were incorporated into the present study. Rats challenged with isoproterenol showed significant heart dysfunction. Treatment with ginsenoside Re blocked the changes in LVEDP, LVSP, and ±dp/dt induced by isoproterenol significantly. These results suggested that ginsenoside Re could ameliorate the impairment in heart function caused by myocardial ischemia.

Adrenergic hormones and their analogs are considered potent stimulants for the fibrosis and hypertrophy of the myocardium [18]. Chronic or acute exposure to isoproterenol induces necrosis and cardiac hypertrophy and promotes fibrosis in animal models. A major contributor to the pathologic features of the fibrosis and hypertrophy of the myocardium is collagen accumulation within the extracellular matrix (ECM) [19]. The latter plays a key part in supporting cardiac-tissue architecture for maintaining the geometry and function of heart chambers.

Collagen in the heart is represented by types I and III, with collagen I impacting greatly on ventricular stiffness due to its tensile and rigid properties [20]. In response to ischemic injury, myocardial fibrosis results from abnormal regulation of the synthesis and degradation of the ECM, leading to accumulation of collagen I. Hydroxyproline is a posttranslational product of proline hydroxylation, and hydroxyproline is thought to be due almost exclusively to collagen [21].

In the present study, administration of isoproterenol (5 mg/kg/day, s.c.) for 7 days induced myocardial hypertrophy in rats. In contrast, rats treated with ginsenoside Re reversed isoproterenol-induced hypertrophic growth markedly, as indicated by a decreased heart weight and HWI. Consistent with evaluation of myocardial hypertrophy, Sirius Red staining and quantitative estimation of collagen clearly showed that isoproterenol exposure induced increased collagen accumulation and fibrosis in rat hearts. However, treatment with ginsenoside Re reduced collagen accumulation and fibrosis formation significantly in the heart. Reduction in hydroxyproline content caused by administration of ginsenoside Re further demonstrated that ginsenoside Re could inhibit isoproterenol-induced collagen accumulation.

TGF-*β*1 is a multifunctional regulator of ECM production, tissue homeostasis, cell organization, and cell division. Disruption of the TGF-*β*1 pathway has been implicated in several human diseases, including myocardial fibrosis [22]. TGF-*β*1 transmits signals predominantly through Smad proteins in the cell cytoplasm. After phosphorylation, Smads translocate to the cell nucleus and act as transcription factors. Activation of the Smad3 cascade increases collagen expression and promotes deposition of fibrous tissue. In the fibroblasts of adults, Smad3 is essential for TGF-*β*1-induced gene expression [23]. mRNA and protein levels of TGF-*β*1 are increased in myocardial-infarction scars and correlate with increased expression of collagen I and Smad3 phosphorylation, suggesting that activation of the TGF-*β*1/Smad3 signaling pathway may contribute significantly to myocardial fibrosis [24]. We showed that isoproterenol administration led to a significant increase in the TGF-*β*1 level, followed by augmentation of Smad3 phosphorylation and collagen-I expression. Treatment with ginsenoside Re decreased the TGF-*β*1 level and also inhibited (at least in part) the increase in Smad3 phosphorylation and collagen-I expression induced by isoproterenol. These findings suggest that the pharmacologic actions of ginsenoside Re to improve isoproterenol-induced myocardial fibrosis and heart failure were associated (at least in part) with regulation of the TGF-*β*1/Smad3 pathway.

Our study had one major limitation. After oral administration, ginsenoside Re is in contact with gastric acids, gastric enzymes, intestinal enzymes, and colonic bacteria. Ginsenoside Re could be metabolized primarily to ginsenoside Rg2 and ginsenoside Rh1 [25]. Studies have shown that ginsenoside Rg2 attenuates the injury due to myocardial ischemia in rats [26]. It has also been reported that ginsenoside Rh1 has effects against isoproterenol-induced cardiotoxicity [10]. Therefore, we could not exclude the possible contribution of metabolites to the protective effects of ginsenoside Re in our study. Studies are needed to evaluate the effect of intravenous administration of ginsenoside Re on myocardial fibrosis and heart failure. Furthermore, Zhang and colleagues demonstrated that ginsenoside Re inhibited the activation of JNK and NF-*κ*B in rats [27]. It also showed that the JNK signaling pathway interacted with the TGF-*β*/SMAD pathway. JNK activation not only augmented TGF-*β* gene transcription, but also induced expression of enzymes that activate the latent form of TGF-*β*. JNK directly phosphorylated Smad3 to enhance transcription of profibrotic molecules [28]. Therefore, the downregulations of TGF-*β*1 and Smad3 caused by ginsenoside Re may be related to the JNK signaling pathway. However, we did not perform a series of experiments silencing TGF-*β*1 or Smad3 to strengthen our conclusion. We openly accept this as another limitation of our study.

## 5. Conclusions

Ginsenoside Re could improve isoproterenol-induced myocardial fibrosis and heart failure in rats. The pharmacologic actions of ginsenoside Re are related (at least in part) to regulation of the TGF-*β*1/Smad3 pathway. These results provide further insight into the molecular mechanisms underlying the protective effects of ginsenoside on ischemic heart diseases.

## Data Availability

The data used to support the findings of this study are available from the corresponding author upon request.
